# Polycationic Glycopolymer Demonstrates Activity Against Persisters and Biofilms of Non-tuberculosis *Mycobacteria* Cystic Fibrosis Clinical Isolates *in vitro*

**DOI:** 10.3389/fmicb.2022.821820

**Published:** 2022-02-21

**Authors:** Vidya P. Narayanaswamy, Stacy M. Townsend, Allister J. Loughran, William Wiesmann, Shenda Baker

**Affiliations:** ^1^Synedgen, Inc., Claremont, CA, United States; ^2^Synspira Therapeutics, Inc., Framingham, MA, United States

**Keywords:** nontuberculous mycobacteria, biofilms, persister cell, antibacterial activity, PAAG

## Abstract

Non-tuberculosis *Mycobacterium* (NTM) is a group of opportunistic pathogens associated with pulmonary infections that are difficult to diagnose and treat. Standard treatment typically consists of prolonged combination antibiotic therapy. Antibiotic resistance and the role of biofilms in pathogen communities, such as NTM persister cells, is an important unmet challenge that leads to increased toxicity, frequent relapse, poor clinical management, and an extended treatment period. Infection recurrence and relapse are not uncommon among individuals with cystic fibrosis (*CF*) or chronic obstructive pulmonary disease (COPD), where thick mucus supports bacterial biofilm production and impairs mucociliary clearance. The study evaluates a membrane-active cationic glycopolymer [poly (acetyl, arginyl) glucosamine (PAAG)] being developed to support the safe and effective treatment of NTM biofilm infections. PAAG shows antibacterial activity against a wide range of pathogenic bacteria at concentrations non-toxic to human epithelial cells. Time-kill curves demonstrated PAAG’s rapid bactericidal potential at concentrations as low as 1X MIC against all NTM strains tested and compared to the standard of care. PAAG treatment prevents persister formation and eradicates antibiotic-induced persister cells in planktonic NTM cultures below the limit of detection (10 colony-forming unit (CFU)/ml). Further, PAAG showed the ability to penetrate and disperse NTM biofilms formed by both rapidly and slowly growing strains, significantly reducing the biofilm biomass (*p* < 0.0001) compared to the untreated NTM biofilms. Microscopical examination confirmed PAAG’s ability to disrupt and disperse mycobacterial biofilms. A single PAAG treatment resulted in up to a 25-fold reduction in live-labeled NTM and a 78% reduction in biofilm thickness. Similar to other polycationic molecules, PAAG’s bactericidal and antibiofilm activities employ rapid permeabilization of the outer membrane of the NTM strains, and subsequently, reduce the membrane potential even at concentrations as low as 50 μg/ml (*p* < 0.001). The outcomes of these *in vitro* analyses suggest the importance of this polycationic glycopolymer, PAAG, as a potential therapeutic agent for opportunistic NTM infections.

## Introduction

NTM (non-tuberculosis *mycobacterium*) are opportunistic human pathogens capable of causing chronic pulmonary infections, particularly in populations with underlying lung diseases, such as cystic fibrosis (*CF*), chronic obstructive pulmonary disorder (COPD), and non-*CF* bronchiectasis (Park et al., 2016; Skolnik et al., 2016; Liu et al., 2018). The incidence of chronic pulmonary infections having NTM as the lead cause is rising, increasing around 8% each year (Mehta and Marras, 2011; Adjemian et al., 2012; Brown-Elliott et al., 2012; Huitt, 2015; Skolnik et al., 2016; Diel et al., 2017, 2018; Martiniano et al., 2017; Park et al., 2017; Jones et al., 2018; Marras et al., 2018). Studies show that 12.7% of US *CF* patients were culture positive with NTM (Cystic Fibrosis Foundation, 2017), and longitudinal data reveal that 19% had one or more NTM species isolated over 4 years (Martiniano et al., 2017). These patients had a higher rate of annual decline in predicted forced expiratory volume (FEV1) than those with no NTM infection (Esther Jr et al., 2010; Huitt, 2015; Skolnik et al., 2016). Pulmonary NTM infections most commonly involve slowly growing mycobacteria (SGM) and rapidly growing mycobacteria (RGM). The most frequently isolated strains are *Mycobacterium avium* complex (MAC; SGM; 76%) and *Mycobacterium abscesses* complex (MABSC; RGM; 18%), which can exist as polymicrobial infections (Gilljam et al., 2004). In North America, 75% of NTM are MAC and 25% are *M. abscesses*. In general, MAC is seen in older people who have more mild disease and *M. abscessus* is associated with more advanced disease and a more rapid decline in lung function. SGM are most often associated with an extended treatment duration (12–15 months) and low cure rates (Ferro et al., 2015). Resistance to commonly used antibiotics often frequently complicate RGM treatment regimens (Ferro et al., 2015).

Successful treatment of NTM in patients and completion of clinical trials are often hindered by the need for combination therapy, resistance to multiple drugs, adverse reactions, and long treatment duration (Griffith et al., 2007; Waters and Ratjen, 2012; El et al., 2013; Jönsson et al., 2013). Antibiotic combination therapy is used and typically includes up to three drugs (macrolides, rifampin, and ethambutol) administered orally, inhaled, and/or intravenously (Renna et al., 2011). Arikayce, a more recently approved inhaled liposomal formulation of amikacin, is specifically designed to target MAC in the lungs (Griffith et al., 2018; ARIKAYCE, 2020). The drug has shown efficacy in a limited and specific population of patients (ARIKAYCE, 2020). As a last resort, NTM infection can require invasive surgical intervention to remove the infection. Since the shift toward macrolide-based multidrug therapy in the 1990s, little progress in antibiotic treatment of NTM has occurred (Griffith and Aksamit, 2012). Treatment of NTM pulmonary infections is further complicated by NTM’s innate antibiotic resistance, slow growth, and ability to form biofilms as well as recalcitrant persister cells. These persisters reflect a state of dormancy that is resilient to antibiotic exposure and can resume growth after removal of the antibiotic or other stressor contributing to the chronicity of infection (Fisher et al., 2017).

NTM dormancy has been demonstrated in various NTM species, particularly in *M. avium*, providing another mechanism of resistance to antibiotic challenge (Archuleta et al., 2005). These non-replicating cultures do not respond to antibiotics because metabolically inactive bacteria are not likely to be killed by traditional antibiotic mechanisms. Most antibiotics target growth-essential functions, so the shift to a metabolically quiescent, non-replicating state is a plausible explanation for the loss of bactericidal activity observed (Lakshminarayana et al., 2015) and the existence of “persister” cells. Most NTM persisters are located intracellularly or in biofilms where nutrients and oxygen concentrations are low and metabolic stressors may contribute to their dormancy (Kostakioti et al., 2013). The existence of persister cells within NTM biofilms is potentially an important cause of treatment relapse and persisters’ ability to enhance the chronicity of NTM infections (Gollan et al., 2019).

PAAG is a class of glycopolymer therapeutics that has demonstrated antibacterial activity against a wide range of Gram-positive and Gram-negative bacteria (Narayanaswamy et al., 2018; Fernandez et al., 2019; Garcia et al., 2022). This polycationic glycopolymer also has been found to increase the efficacy of existing therapies against drug-resistant clinical isolates of *Burkholderia cepacia* and clinical multidrug-resistant *Staphylococcus aureus* (MRSA) isolates (Narayanaswamy et al., 2017, 2018). In addition to potentiating antibiotics, previous studies show PAAG treatment prevents the formation of persister cells in clinical isolates of *Pseudomonas aeruginosa* and eradicates antibiotic-induced persisters (Narayanaswamy et al., 2018). Furthermore, *in vitro* studies have demonstrated PAAG’s ability to disperse and permeabilize mature biofilms formed by multidrug drug-resistant *P. aeruginosa*, which significantly removes biofilm biomass and the viable bacteria present in the biofilm (Garcia et al., 2022). Apart from PAAG’s ability to facilitate rapid biofilm removal, studies have also demonstrated its potential to increase mucociliary clearance *in vivo* (Fernandez et al., 2019; Narayanaswamy et al., 2019). PAAG is currently being examined in dose-ranging studies as an inhaled treatment in Europe. Currently, all tested doses have been shown to be safe and tolerable.

While we have shown PAAG to be effective against the Gram-negative *Pseudomonas aeruginosa*, that is insufficient to suggest efficacy against non-tuberculous mycobacteria (NTM). Non-tuberculous mycobacteria (NTM) are a different class of bacteria from previously tested organisms, with its lipid-rich impermeable outer membrane, complex growth cycles, dormancy, and adaptation. The presence of a long chain lipid and waxy outer membrane is the major determinant of their physiology, growth, ecology, and epidemiology reduces the transport rate though the outer membrane. The difference in the membrane structure and growth period varied from strain to strain, this made working with NTM challenging. Further, NTM is not susceptible to many of the antibiotics that treat Gram-negative or Gram-positive bacteria. Despite the substantial variations in drug susceptibility among NTM interspecies, treatments are combined yet ineffective, often leading to antibiotic tolerance and persistence. Furthermore, the fact that from 2010 to 2016, 20% of people with *CF* grew NTM at least once, made it eminent to learn and understand NTM and the effect of PAAG on these bacteria.

The current study evaluates PAAG’s antibacterial and anti-biofilm potential against clinical NTM isolates, including fast and slow-growing species isolated from *CF* patients. The concentration-dependent and time-dependent bactericidal activities of PAAG were examined by generating time-kill curves compared to antibiotics used as a standard of care. NTM persisters were isolated using modified time-kill method (Moreira et al., 2016; Mukherjee et al., 2016). Eradication of NTM persisters by PAAG alone and in combination with standard of care antibiotics was evaluated. Further characterization of activity examined the permeabilization of the NTM membrane by PAAG relative to standard antibiotic treatments. Static biofilm assays and minimum bactericidal eradication concentration (MBEC) assays were used to evaluate the ability of PAAG to disrupt preformed NTM biofilms, along with the visual confirmation using confocal laser scanning microscopy (CLSM) and scanning electron microscopy (SEM). Furthermore, the evaluation of PAAG’s ability to potentiate antibiotics against the SGM and RGM NTM isolates existing in biofilm state was performed.

## Materials and Methods

### Bacterial Strains and Culture Conditions

Six *CF*-relevant isolates of *Mycobacteria*, including *M. abscessus subsp. Abscessus* (MABSC) (NTM 0082, NTM 0079), *M. abscessus subsp. bolletii* (NTM 0003), *M. gordonae* (NTM 0274), and *M. avium complex* (NTM 0260, NTM 0813), and one non-*CF*, *Mycobacterium intracellulare* (NTM 0125) were acquired from Cystic Fibrosis Foundation (CFF) *Burkholderia cepacia* Research Laboratory and Repository (John J. LiPuma). The bacterial strains acquired included both rapid-growing and slow-growing [*M. avium complex* (MAC) (NTM 0260)] *Mycobacterium CF* isolates. These isolates were recovered from both male and female patients over a broad age range (10–76 years) in the United States in 2014.

The bacteria were grown in 7H9 Middlebrook broth and stored at −80° freezer with 15% glycerol and recovered from frozen stock on 7H10 Middlebrook (MB) agar overnight at 30°C. Bacterial colony counts were also grown on MB agar (Difco). Vehicle control solutions used, glycerol (Spectrum), NaCl (Sigma), and dornase alfa (Sigma) were pharmaceutical grade (USP). The treatment concentrations of hypertonic saline (7%) and dornase alfa (3.2 μg/ml) used were biologically relevant (Reeves et al., 2012). Antimicrobial’s rifampicin (RIF; Sigma), amikacin (AMK; TCI), azithromycin (AZM; TCI), ethambutol (E; Sigma), ciprofloxacin (CIP; Sigma) was used in the study. Stock solutions and dilutions were prepared fresh.

### PAAG Glycopolymer

The polycationic proprietary glycopolymer is an arginine derivative of a natural polysaccharide poly-N-acetyl-glucosamine (PAAG) and is polycationic and soluble at physiologic pH. PAAG has been shown to be safe in Phase 1 clinical studies as SNSP113 and is currently in Phase 2 clinical studies.

### Antimicrobial Testing

The minimum concentration at which no growth was observed visually was considered as the MIC for the condition (Wiegand et al., 2008). The experiments were repeated three times with three replicates (*n* = 3).

### Time-Kill Analysis

Representative strains from the slow-growing and rapidly growing mycobacteria strains, MAC (NTM 0260) and MABSC (NTM 0079) were grown and resuspended in Middlebrook 7H9 broth to obtain a bacterial culture of 10^6^–10^7^ CFU/ml. Then, the inoculum was treated with PAAG, RIF, AMK, AZM, and E at concentrations 1X, 2X, and 4X MIC in Middlebrook 7H9 broth containing 1.38% glycerol and incubated at 30°C. Wells with NTM and no antimicrobials were used as controls. After incubation, culture aliquots were sampled at 0, 1 h, and on days 2, 4, 6, 10, and 14 for MAC strain and days 1, 2, and 4 for the MABSC strain, respectively. The aliquots were centrifuged at 13,000 rpm for 1 min, washed with 1XPBS, and resuspended in 7H9 media prior to serial dilution and plating on 7H10 agar plates. The plates were incubated at 30°C for 4–10 days. The NTM viability was determined by enumerating colonies growing on 7H10 MB agar plates (CFU/ml). Bactericidal activity was defined as a 3-log reduction of viable counts in culture treated with PAAG compared with that of the vehicle-treated control at a specific time point. Experiments were conducted with three independent cultures of each strain, with three replicates within each experiment (*n* = 3). Limit of detection- 10 CFU/ml.

### Outer Membrane Permeability and Inner Membrane Depolarization

The extent of membrane permeabilization was assessed using propidium iodide (PI) dye (Helander and Mattila-Sandholm, 2000). All assays were performed at room temperature. Bacterial cultures were grown up to 0.5 OD. Then, PI (17 μg/ml) was added to the wells of a 96-well plate containing bacterial culture and fluorescence was measured *via* SpectraMax Gemini XPS (Molecular Devices). Antibiotics or PAAG were prepared at concentrations 1X and 4X times their MIC and added to the wells containing the mixture. Cells treated with 0.1% Triton X-100 were used as a positive control. Negative controls included PI alone and PI on cells. The plates were mixed thoroughly prior to obtaining fluorescence measurements. Fluorescence was measured at excitation and emission wavelengths of 535 and 625 nm, every 10 min up to 4 h. The experiment was performed in triplicate with three independent cultures (Padwal et al., 2015). Experiments were conducted with three independent cultures of each strain, with three replicates within each experiment (*n* = 3).

Changes in the potential across the cell membrane were quantified by the 3,3′-diethyloxacarbocyanine iodide (DiOC2) dye (Foss et al., 2013). Cells were grown till 0.5 OD and then subjected to treatments at concentrations 4X their MIC for 1 h. Post-treatment samples were centrifuged and resuspended in 5 mM HEPES (with 0.5 M EDTA) with 30 μM DiOC2. Samples were further incubated in dark for 1 h at 30°C with aeration at 200 rpm. Post-incubation, 200 μl of each sample was aliquoted into a 96 well plate and the fluorescence was detected by excitation at 620 nm and emission at 670 nm, respectively. Data were plotted as relative fluorescence units (RFU) for each sample. Experiments were conducted with three independent cultures of each strain, with three replicates within each experiment (*n* = 3).

### NTM Persisters

The method used to isolate the persisters was adapted from Hu et al. (2000) bacterial culture models. All tests were carried out in triplicates and repeated at least two times with two independent cultures.

#### Bacterial Culture Conditions

Working stock of the MABSC (NTM 0079) and MAC (NTM 0260) strains was produced by culturing the bacteria on 7H10 agar supplemented with oleic acid–albumin–dextrose–catalase complex (referred to as 7H10 agar plates). Single colony was picked and propagated in 7H9 media containing 0.05% Tween 80 supplemented with 10% (wt./vol) albumin–oleic acid–dextrose–catalase complex (referred to as 7H9 broth).

#### Bacterial Growth Conditions

For the study, bacterial cultures were initially grown overnight at 30°C 7H9 broth. The cultures were then centrifuged at 13,000 rpm for 5 min. The pellet was washed with 1XPBS and resuspended to obtain a bacterial concentration of 10^8^ CFU per ml in 7H9 broth and further diluted to 10^6^ cells/ml for the experiment. Serial dilution (10^−7^ (20:200) using sterile water in a 96-well plate) from each bacterial suspension (100 μl) was plated in duplicate (2X20μl) onto 7H10 agar plates and CFU/ml were calculated following overnight incubation at 30°C (limit of detection- 10 CFU/ml). The bacterial cultures were grown in 7H9 broth for 4 days (MABSC) and 14 days (MAC).

#### Sonication

To obtain evenly dispersed bacteria prior to experimental treatment, any sedimented clumps of bacteria were broken up by sonicating for 5 min, in a water bath sonicator. The sonication phase was optimized for each strain tested and sonicating for 5 min using a water bath sonicator does not result in loss of cell viability.

#### Antimicrobial Preparation

Antibiotics, namely, rifampicin, amikacin, ciprofloxacin, ethambutol, was prepared freshly at concentration 4X MIC and PAAG at concentration 1XMIC. All the antimicrobials were dissolved in 7H9 broth.

#### Experimental Setup

Five milliliter of diluted bacterial culture was added to five sets of culture tubes, each set containing four tubes.

The antimicrobial prepared was added at final concentration, to all sets and the tubes were incubated at 30°C for 5 days for MABSC and 14 days for MAC. Aliquots (100 μl) were sampled from all sets on days 0, 1, 2, 3, 4, and 5 for MABSC and on days 0, 2, 4, 5, 6, 10, and 14 serially diluted and spot plated in triplicates onto 7H10 plates to obtain viable counts.

The second set of tubes was further incubated for 7 days (recovery phase). Briefly, the bacterial culture in the tubes was pelleted by centrifuging at 13,000 rpm for 1 min washed with 1XPBS, resuspended in 7H9 broth prior to incubation. Aliquots (100 μl) were sampled, serial diluted, and spot plated in triplicates on days 6, 8, 10, and 12 for MABSC and 16, 18, and 21 for MAC to obtain the viable counts.

The bacterial cultures in the third and fourth set of tubes were pelleted by centrifugation at 13,000 rpm for 1 min and resuspended in 7H9 media. Antibiotics and PAAG, respectively, were added to the bacterial cultures in set three and four at the end of the 7-day recovery phase and incubated for 5 days at 30°C for MABSC and 14 days for MAC. Post-incubation the bacterial culture in the tubes was pelleted by centrifuging at 13,000 rpm for 1 min washed with 1XPBS, resuspended in 7H9 broth, and incubated at 30°C for 7 days (MABSC) and 21 days (MAC). Aliquots (100 μl) were sampled, serial diluted, and spot plated on days 12, 14, 16, 17, 18, 20, 22, and 24 for MABSC and 22, 24, 25, 26, 28, 30, 32, 36, 39, 45, 47, 50, 54 and 56 for MAC cultures to obtain viable counts.

The bacterial cultures in the fifth set of tubes were pelleted by centrifugation at 13,000 rpm for 1 min, washed, and resuspended in 7H9 media. Antibiotics and PAAG were added to the bacterial culture at the end of the 7-day (MABSC) and 14-day (MAC) recovery phase and incubated at 30°C for 2X 5-day treatments (MABSC)/2X 7-day treatments (MAC). This was followed by a 7-day (MABSC) and 21-day (MAC) recovery phase. Aliquots (100 μl) were sampled, serial diluted, and spot plated on days 14, 16, 17, 18, 20, 22, 24, 26, and 28 for MABSC and on days 22, 24, 25, 26, 28, 30, 32, 36, 39, 42, 45, 47, 50, 54, and 56 for MAC to obtain viable counts.

### Effect of PAAG Against Preformed Biofilms

Five NTM isolates were acquired from CFF *Burkholderia cepacia* Research Laboratory and Repository (John J. LiPuma). Bacteria were grown overnight in Middlebrook 7H9 broth. The overnight grown bacteria were adjusted to 1 McFarland turbidity standard and further diluted 1:30 in Middlebrook 7H9 broth. The diluted culture was added to the 96-well plate. The NTM biofilms were grown for 1–4 weeks, at 30°C, without shaking, depending on the *Mycobacterium* strain. Cultures were frequently sampled and stained for contamination. Biofilms were gently rinsed twice and treated with PAAG (50–200 μg/ml) formulated in 1.38% glycerol, pH 7.4, and incubated at room temperature for an hour. After an hour, the biofilms were rinsed again in 1XPBS and dried for 2 h at 30°C. The biofilms were then stained with 1% crystal violet for 15 min. Stained biofilms were rinsed twice with 1XPBS and eluted in absolute methanol. After incubation for 5 min, the solubilized crystal violet was transferred onto a fresh microtiter plate and the OD was read at 600 nm. The average absorbance of biofilm-forming isolates was greater than the average absorbance of the negative control wells ± 3 SEM, confirmed biofilm formation.

### Live-Dead Staining of the NTM Biofilms

The BacLight LIVE/DEAD bacterial viability kit (Molecular probes, Eugene, OR, United States) was used to stain the NTM biofilms. The bacteria were grown on 0.18 mm glass coverslips overnight in 7H9 Middlebrook broth and then adjusted to 1 McFarland turbidity standard (Pfyffer et al., 2003). The bacterial culture was further diluted 1:30 in 7H9 Middlebrook broth and seeded into each well of a 12-well tissue culture plate. The cover slips were gently placed into each well, at a 90° angle relative to the bottom of the wells so that the meniscus of the medium was at the center of the coverslip. The biofilm grew on the coverslip for 1–4 weeks, depending on the strain, at 30°C. The non-adherent cells were removed following a gentle rinse with 1XPBS. The biofilms were treated with PAAG (50–200 μg/ml) for 1 h, rinsed, and stained. The LIVE/DEAD stain was prepared according to manufactures’ instructions. The coverslips were washed to remove any excess stain and observed with a 63X lens objective by CSLM using a TCS SP5 microscope and software (Leica) with an excitation at 488 nm and emission detected using a dual-band emission filter (500–550 nm/598–660 nm). To obtain the depth of the biofilm, z-stack images were attained at a distance of 0.5 μm as described in previous studies (Pfyffer et al., 2003). At least 45 depths of the biofilm were measured for each image captured, in every experiment conducted. A total of three images of five separate views were taken for each cover slip. Data were represented as the mean of all the images was used to calculate the thickness of the biofilm.

### Scanning Electron Microscopy

For scanning electron microscopy (SEM) imaging, biofilms were grown for 24 h on silicon wafers placed in sterile 12-well plates. PAAG at a concentration of 200 μg/ml was added and the plate was incubated for 1 h. The biofilms were fixed overnight with 2.5% glutaraldehyde and 0.5% paraformaldehyde in 0.1 M phosphate buffer, followed by rinsing with 0.1 M phosphate buffer (3 × 10 min each). The biofilms were then dehydrated gradually by being washed sequentially with 10, 25, 50, 75, and 95% alcohol (5 min each) and 100% alcohol (3 × 5 min each). Hexamethyldisilizane (HMDS) was used for overnight drying. Samples were then sputter-coated with gold prior to scanning electron microscopy imaging.

## Results

### Antimicrobial Resistance Profiling

NTM isolates were characterized using Clinical and Laboratory Standards Institute (CLSI) guidelines to assess their susceptibility to the antimicrobials (Table 1). All the NTM isolates tested were found to be resistant to rifampicin and ethambutol with an MIC greater than their clinical breakpoints (Table 1). All *M. abscessus* strains tested were also found to resistant to azithromycin and ciprofloxacin to a level greater than their breakpoint MICs. Apart from *M. abscessus subsp. bolletti*, the other *M. abscessus* strains were also found to be resistant to amikacin according to CLSI standards (Table 1). All the *M. avium* strains tested were determined to be sensitive to amikacin and ciprofloxacin and showed intermediate resistance to azithromycin (Table 1). *Mycobacterium gordonae* and *M. intracellulare* strains were sensitive to amikacin and ciprofloxacin and showed resistance or intermediate resistance to azithromycin (Table 1). The strains had PAAG MICs between 16 and 250 μg/ml.

**Table 1:** Minimal inhibitory concentrations (MICs) of antimicrobials tested against MDR-Mycobacteria isolates, isolated from *CF* patients.

Repository number	Species	Source	MIC (μg/ml)					
			PAAG	RIF	E	AMK	AZM	CIP
NTM0260	*M. avium complex*	*CF*	16	4 (R)	2 (R)	2 (S)	2 (I)	0.125 (S)
NTM0813	*M. avium complex*	*CF*	16	4 (R)	2 (R)	2 (S)	2 (I)	0.125 (S)
NTM0003	*M. abscessus subsp. bolletii*	*CF*	128	8 (R)	8 (R)	4 (S)	32 (R)	16 (R)
NTM0079	*M. abscessus subsp. abscessus*	*CF*	250	2 (R)	32 (R)	8 (R)	32 (R)	8 (R)
NTM0082	*M. abscessus subsp. abscessus*	*CF*	250	2 (R)	32 (R)	8 (R)	32 (R)	8 (R)
NTM0274	*M. gordonae*	*CF*	32	2 (R)	2 (R)	<0.0625 (S)	2 (I)	<0.0625 (S)
NTM0814	*M. intracellulare*	non-*CF*	16	4 (R)	2 (R)	<0.0625 (S)	4 (R)	<0.0625 (S)

*Clinical breakpoints according to CLSI standards were as follows; ≥1 μg/ml (R) for rifampicin (RIF); ≥2 μg/ml (R) for ethambutol (E), ≤16 μg/ml (S), 32 μg/ml (I), ≥64 μg/ml (R) for amikacin (AMK); ≥8 μg/ml (R), 4 μg/ml (I), ≤2 μg/ml (S) for azithromycin (AZM), ≥4 μg/ml (R), 2 μg/ml (I), ≤1 μg/ml (S) for ciprofloxacin (CIP). R, resistant strains; S, sensitive strains; and I, intermediate.*

### PAAG Exhibited Bactericidal Activity Against Both Rapidly Growing and Slow-Growing NTM Isolates

**Figure 1:**
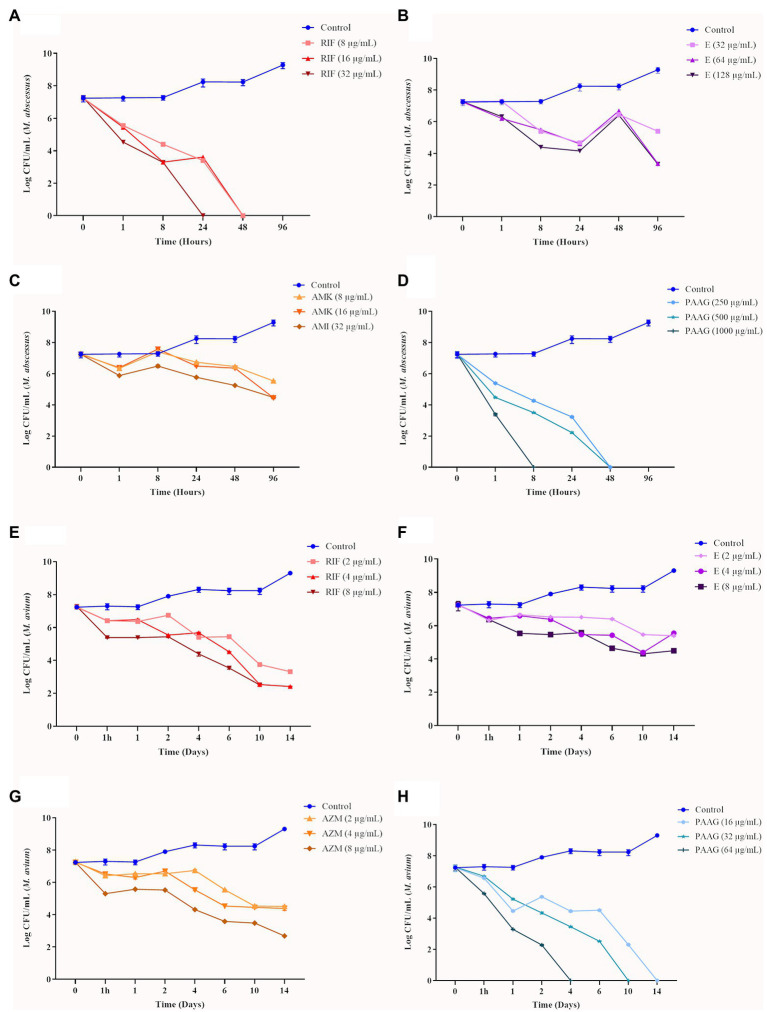
Antimicrobial efficacy of poly (acetyl, arginyl) glucosamine (PAAG) or antibiotics against rapid and slow-growing non-tuberculosis *Mycobacterium* (NTM) isolates. Time-kill curves show the sensitivity of MABSC (NTM 0079) **(A–D)** and MAC (NTM 0260) **(E–H)** to PAAG and the antibiotics tested, represented as CFU/ml at various points of time. The antimicrobial was added at time point 0 after and monitored for 4–14 days, depending on the NTM isolate tested. Data represent the mean of three independent experiments (*n* = 3 each experiment) ± SEM.

Time-kill curves were generated for the representative rapid (MABSC) and slow-growing (MAC) *Mycobacterium* clinical isolates tested (Figures 1A–H). Bactericidal activity of PAAG resulted in a rapid decrease in CFU, following eradication of both rapid and slow NTM strains tested (Figures 1D,H). PAAG completely eradicated the MABSC population within 8–48 h of treatment and MAC within 4–14 days of treatment in a dose-dependent manner (Figures 1D,H). Rifampicin was observed to be bactericidal against rapid and slow-growing NTM, eradicating MABSC in a dose- and time-dependent manner and eradicating MAC at 4X MIC (Figures 1A,E). Amikacin was found to be bacteriostatic against MABSC, as the log CFU/ml over time remained the same as the starting log CFU/mL (Figure 1C). *Azithromycin and ethambutol demonstrated moderate decrease in CFU/mL of MAC strain at 4X concentrations tested* (Figure 1G). *Ethambutol was found to be bactericidal against MABSC*, resulting in a 3-log reduction of the viable bacteria (Figure 1B).

### PAAG Permeabilizes the Outer Membrane of Both Rapid and Slow-Growing NTM and Depolarizes the Cytoplasmic Membrane

**Figure 2:**
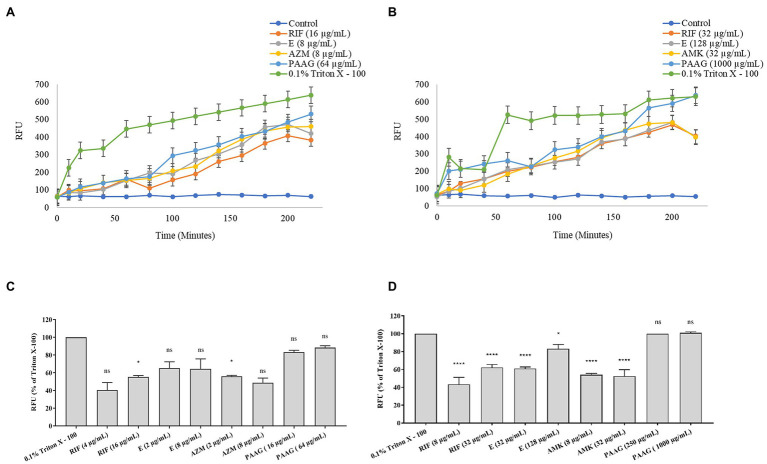
PAAG permeabilizes the outer membrane and depolarizes inner membrane of NTM. **(A,B)** PAAG’s ability to permeabilize the outer membrane of *Mycobacterium avium* complex (MAC; NTM 0260) **(A)** and *Mycobacterium abscesses* complex (MABSC; NTM 0079) **(B)** was assessed. An increase in fluorescence is indicative of pore formation in bacterial membrane. **(C,D)** Membrane depolarization activity by antimicrobials on MAC (NTM 0260) **(C)** and MABSC (NTM 0079) **(D)**. Data representative of three independent experiments (*n* = 3 each experiment) and shown as mean ± SEM. Statistical analysis was done using Dunnett’s multiple comparison tests compared to the positive control. *p < 0.05; ****p < 0.0001; ns, no significant difference compared to the positive control.

PAAG at concentrations 1X and 4X MIC resulted in a dose and time-dependent permeabilization of the rapid and slow-growing NTM strains (Figures 2A,B). Antibiotics treatment resulted in a moderate increase in the RFU relative to PAAG and positive control (Figures 2A,B).

Figures 2C,D shows the depolarization of the cytoplasmic membrane (RFU < 80%) by PAAG, with a more rapid permeabilization of the NTM membranes compared to the other antibiotics tested. Treatment with rifampicin resulted in a dose-dependent increase in RFU achieving <60% depolarization, compared to PAAG and the positive control against MABSC and MAC. Treatment with ethambutol resulted in a dose-dependent increase in RFU, attaining 80% depolarization at 4XMIC against MABSC and MAC strains tested. Treatment with amikacin and azithromycin resulted in a stable RFU for both MABSC and MAC, despite of the increasing concertation’s of the antibiotic tested.

### PAAG Treatment Eradicates Both Rapid and Slow-Growing NTM, Preventing the Formation of Persisters

**Figure 3:**
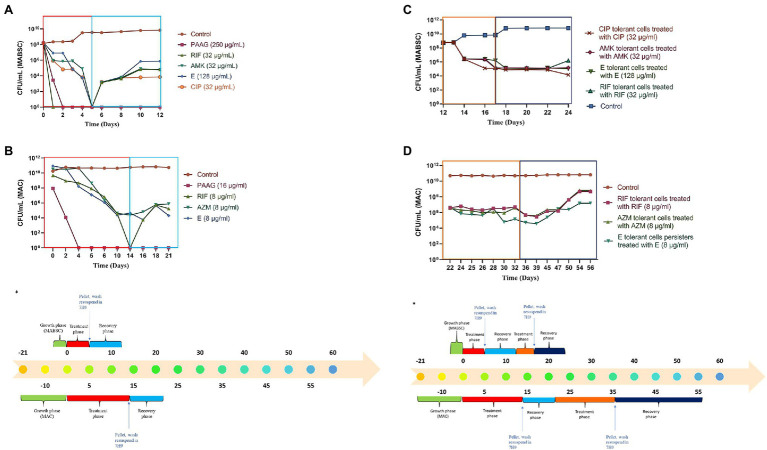
**(A,B)** PAAG eradicates viable bacteria in rapid and slow-growing NTM cultures eliminating possibilities of regrowth. Time-kill curves generated for MABSC (NTM 0079), and MAC (NTM 0260) treated with PAAG/antibiotics emphasized PAAG’s potential to eradicate viable NTM, averting any possibilities of regrowth in antimicrobial-free media. Antibiotic concentrations used are indicated on the legend provided on the right side of the graph. **(C,D)** NTM persister formation, induced by the antibiotics, survived further treatments with same antibiotic. **(A)** C-MABSC (NTM 0079). **(B)** D-MAC (NTM 0260). Data representative of three independent experiments (*n* = 3 each experiment) and shown as mean ± SEM. *Experimental timeline. The colored boxes indicate the respective phase described in the experimental timeline.

Previous studies have shown that NTM cultures are capable of surviving nutrient starvation without significant loss of viability (Fernandez et al., 2019). PAAG treatment resulted in rapid decline in viable bacteria leading to eradication of viable bacteria in MABSC and MAC cultures without regrowth or formation of persisters (Figures 3A,B). Rifampicin treatment also led to rapid decline in MABSC and MAC cultures resulting in eradication of viable MABSC and MAC cultures; however, both MABSC and MAC cultures regrew in antibiotic-free media (Figures 3A,B). The bimodal growth pattern exhibited by these rapid and slow-growing mycobacterial strains in response to antibiotics confirms the existence of persister cells (Novo et al., 2000; Yam et al., 2020). Amikacin, ethambutol, and ciprofloxacin were bactericidal against MABSC, leading to complete eradication of viable bacteria, followed by regrowth in antibiotic-free media (Figure 3A). Azithromycin and ethambutol exhibited bactericidal effect against MAC, reducing the starting log CFU/ml by greater than 3 logs but failed to completely eradicate the bacterial population (Figure 3B).

The MABSC and MAC persisters formed in response to antibiotic treatments were subjected to a recovery phase where the cells were washed and resuspended in an antibiotic-free media for 7 days. Post-recovery phase these cells were treated further with the same antibiotic at similar concentrations for to study the effect of the antibiotics on MABSC and MAC persister cells (Figures 3C,D). The antibiotic treatments failed to show any effect on the MABSC and MAC persister populations (Figures 3C,D). The cells were found to be dormant during the treatment phase. Following the treatment these cells were washed and resuspended in antibiotic-free media for 7 (MABSC) and 21 (MAC) days. There was no significant increase or decrease in growth observed during this recovery phase.

### PAAG Treatment Eradicates NTM Persisters

**Figure 4:**
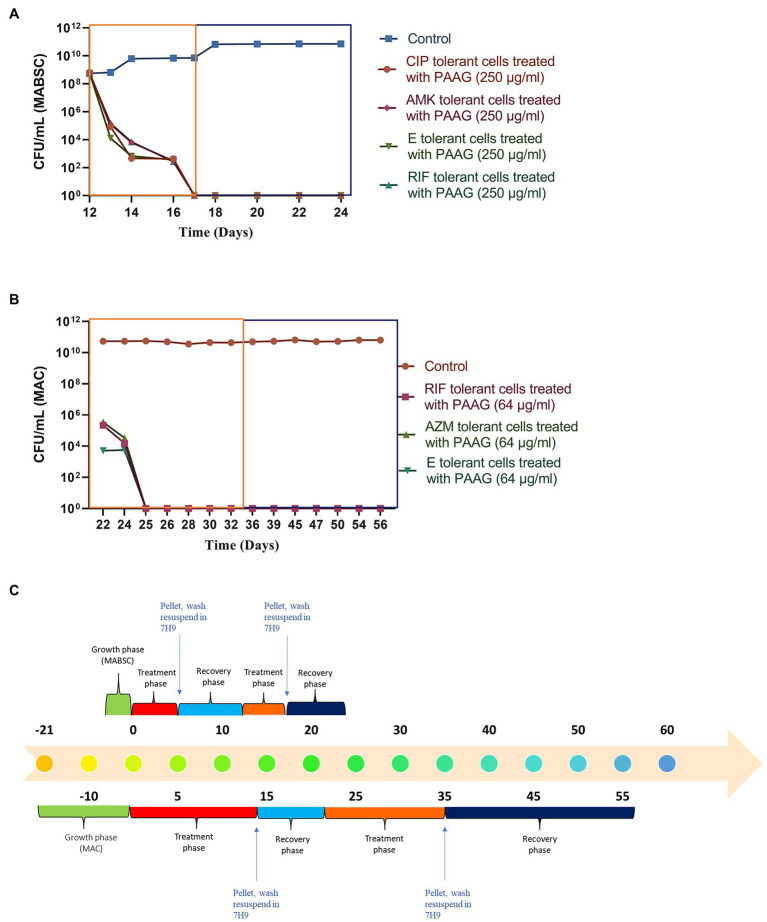
**(A,B)** PAAG treatment eradicates NTM persisters. Treatment with PAAG eradicated the dormant cell population that persisted in MAC and MABSC cultures. No regrowth was observed for up to 7 [MABSC (NTM 0079)] and 21 [MAC (NTM 0260)] days in antibiotic-free media. Data representative of two independent experiments (*n* = 3 each experiment) and shown as mean ± SEM. **(C)** Experimental timeline. The colored boxes indicate the respective phase described in the experimental timeline.

Post-antibiotic treatment and the consecutive recovery phase in antibiotic-free media, the subpopulation of tolerant MABSC and MAC cells left behind, were treated with PAAG for 7 (MABSC) and 14 (MAC) days. PAAG treatment was associated with a rapid decline in the MABSC and MAC persister cells, followed by their complete eradication (Figures 4A,B). No regrowth observed in neither MABSC nor MAC cultures, when washed and resuspended in antimicrobial-free media for the entire duration of the recovery phase, that is, 7 (MABSC) and 21 (MAC) days.

### PAAG’s Activity Was Uninterrupted by the Presence of Antibiotics

**Figure 5:**
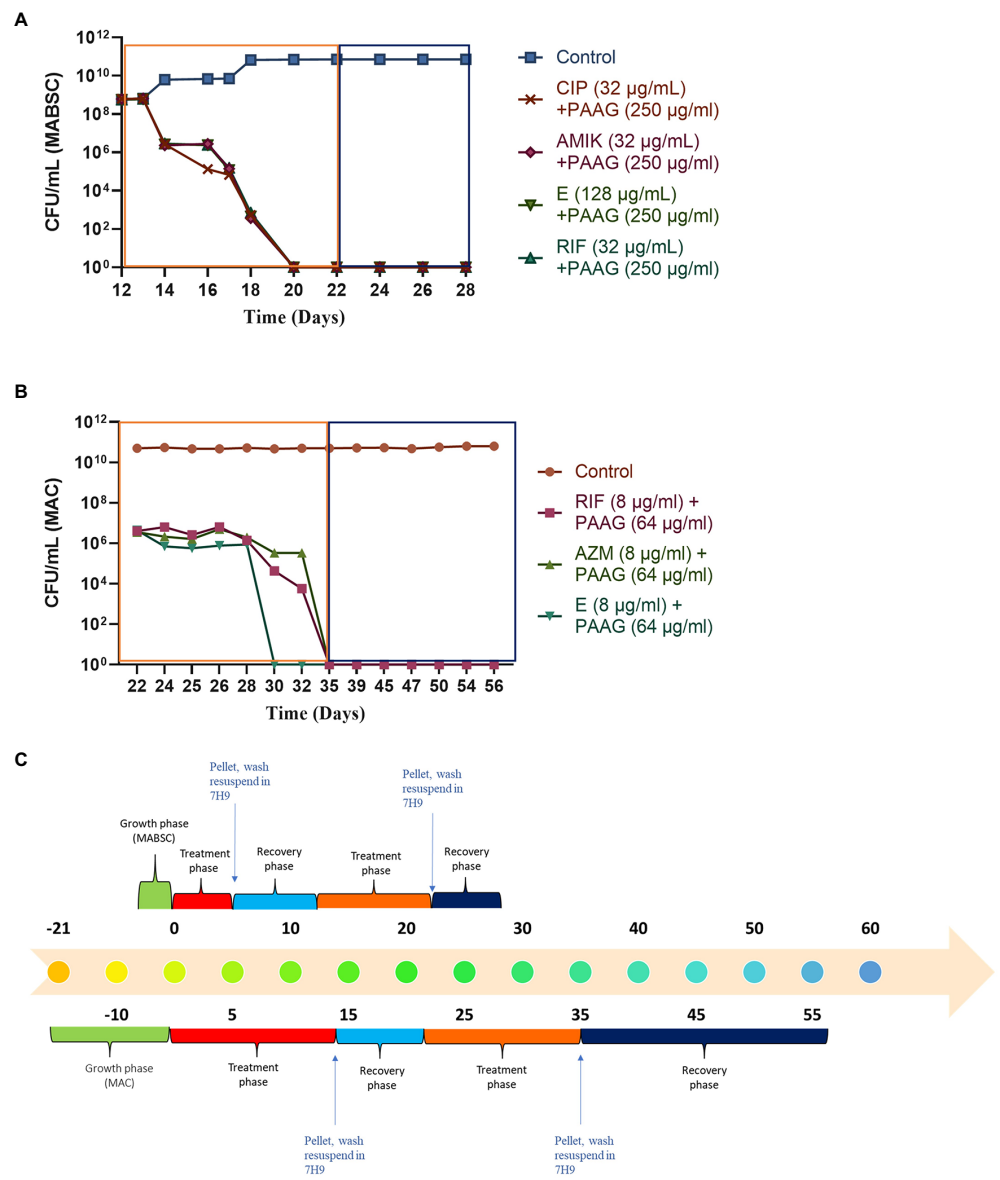
**(A,B)** PAAG eradicates NTM persisters even in the presence of antibiotics. PAAG treatment in addition to antibiotic treatment resulted in complete eradication of the persistent subpopulations present in the MABSC and MAC cultures. No regrowth observed when the cultures were washed and resuspended in antimicrobial-free media. Data representative of two independent experiments (*n* = 3 each experiment) and shown as mean ± SEM. **(C)** Experimental timeline. The colored boxes indicate the respective phase described in the experimental timeline.

The study also assessed the ability of PAAG to work in parallel with the antibiotics used in the standard of care. This step was important as it shed light into better understanding PAAG’s potency in the presence of these antibiotics. Post-antibiotic treatment and recovery in antibiotic-free media the tolerant population in MABSC and MAC cultures were subjected to the same concentrations of antibiotics for 5 days (MABSC) or 7 days (MAC) followed by 5 days (MABSC) or 7 days (MAC) of PAAG treatment. Antibiotics had no effect on the NTM persister cells as shown in Figures 5A,B. Addition of PAAG resulted in a rapid decline in the number of persister cells in both MAC and MABSC cultures (Figures 5A,B). The results were indicative of the potency of PAAG in eradicating the MABSC and MAC persister cells (Figures 5A,B). No regrowth was observed in MABSC and MAC cultures up to 7 and 21 days post-resuspension in antimicrobial-free media. PAAG’s ability to eradicate MABSC and MAC persister cells in the presence of antibiotics (Figures 5A,B) indicates that its activity was not affected by the presence of the antibiotics tested.

### PAAG Permeabilizes MAC and MABSC Biofilms

**Figure 6:**
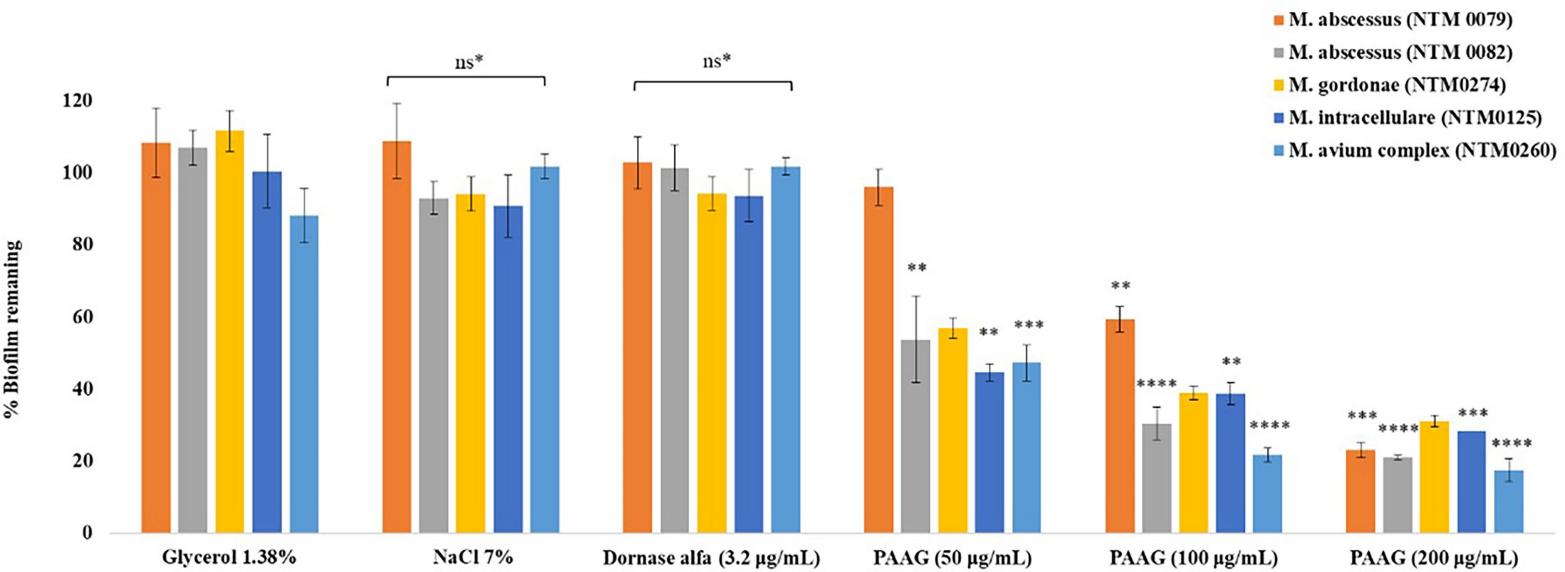
PAAG treatment leads to significant decrease in NTM biofilm biomass. Decrease in biofilm biomass was quantified using crystal violet staining in a static TCP biofilm assay. Data is the (%) biofilm remaining ± SEM. Statistical analysis was done using Dunnett’s multiple comparison tests compared to the untreated control. ** *p* < 0.01; *** *p* < 0.001; **** *p* < 0.0001. ns*, data not significantly different from the 1.38% glycerol and the untreated control.

PAAG treatment resulted in a dose-dependent reduction in preformed biofilms, suggested by rapid reduction in the biofilm biomass, following 1-h treatment (Figure 6). PAAG treatment for an hour at a concentration 200 μg/ml resulted in 85% reduction of preformed MAC biofilms (*p* < 0.0001) and up to 80% reduction of preformed *M. abscessus* complex (*p* < 0.0001), *M. intracellulare* (*p* < 0.001) and *M. gordonae* biofilms (Figure 6). NTM0079 is a highly resistant *CF* isolate, resistant to most antibiotics tested, and explains the higher dose of PAAG required to disrupt and permeabilize the biofilm formed by this strain. Both hypertonic saline and dornase alfa had no significant influence on preformed biofilm disruption compared to 1.38% glycerol treated and the untreated NTM biofilms.

### Visualization of PAAG Treated NTM Biofilms *via* Confocal Laser Scanning Microscope

**Figure 7:**
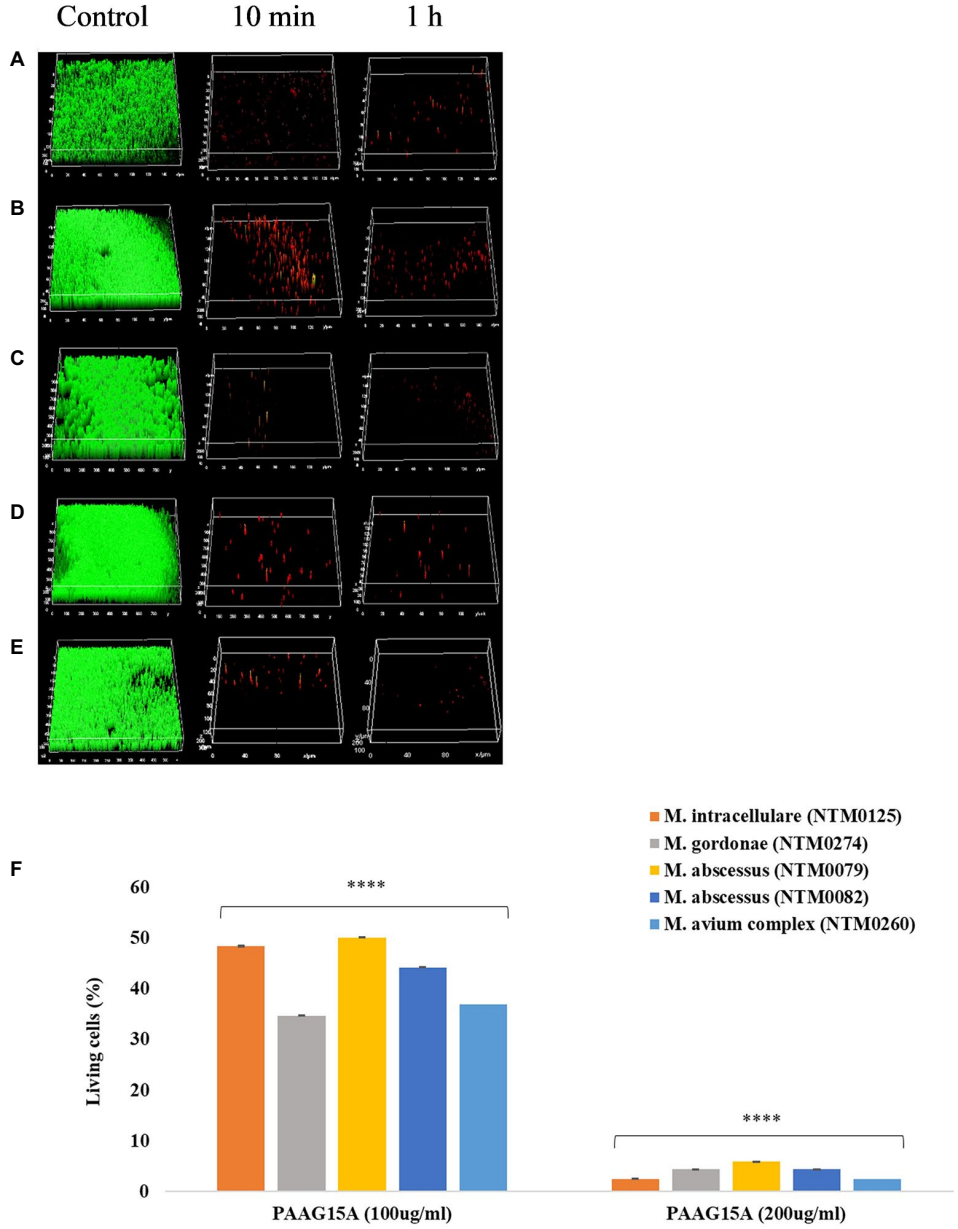
**(A–F)** PAAG disrupts mature NTM biofilms. Representative images of LIVE/DEAD stained Bcc biofilms treated with 200 μg/ml of PAAG for 10 min or 1-h visualized by CLSM. **(A)** *M. intracellulare* NTM0125, **(B)** *M. abscessus* complex NTM0079, **(C)** *M. abscessus* complex NTM0082, **(D)** *M. gordonae* NTM0274, and **(E)** *M. avium complex* NTM0260. (Green = live; Red = dead). Scale bar = 10 μm. The average thickness measurement for the control biofilms was found to be 42.6 μm. **(F)** CLSM was used to measure the number of LIVE-labeled (SYTO 9 labeled) bacteria following 1-h treatment with PAAG. Statistical analysis was done using Dunnett’s multiple comparison tests compared to the untreated control. **** *p* < 0.0001.

NTM biofilms were grown on glass coverslips for 1–6 weeks, depending on the strain, and exposed to vehicle control (1.38% glycerol, pH 7.4) and PAAG (50–200 μg/ml) prior to staining [SYTO 9/Propidium iodide (LIVE/DEAD)] and visualization. Figures 7A–E shows three-dimensional confocal images obtained for NTM biofilms treated with PAAG for 10 min and 1 h compared to the vehicle-treated control. Significant biofilm disruption occurred within 10 min of treatment with 200 μg/ml PAAG compared to vehicle control (Figures 7A–E). Quantitative analysis of the percentage of LIVE cells for the 1 h treatment is shown in Figure 7F. While there were strain-specific differences all five NTM isolates showed a significant reduction. A 19- to 25-fold reduction in live NTM was observed for all rapid and slow-growing NTM clinical isolates tested, following treatment with 200 μg/ml of PAAG for 1 h Figure 7F.

### PAAG Treatment Results in Significant Reduction in NTM Biofilm Thickness

**Figure 8:**
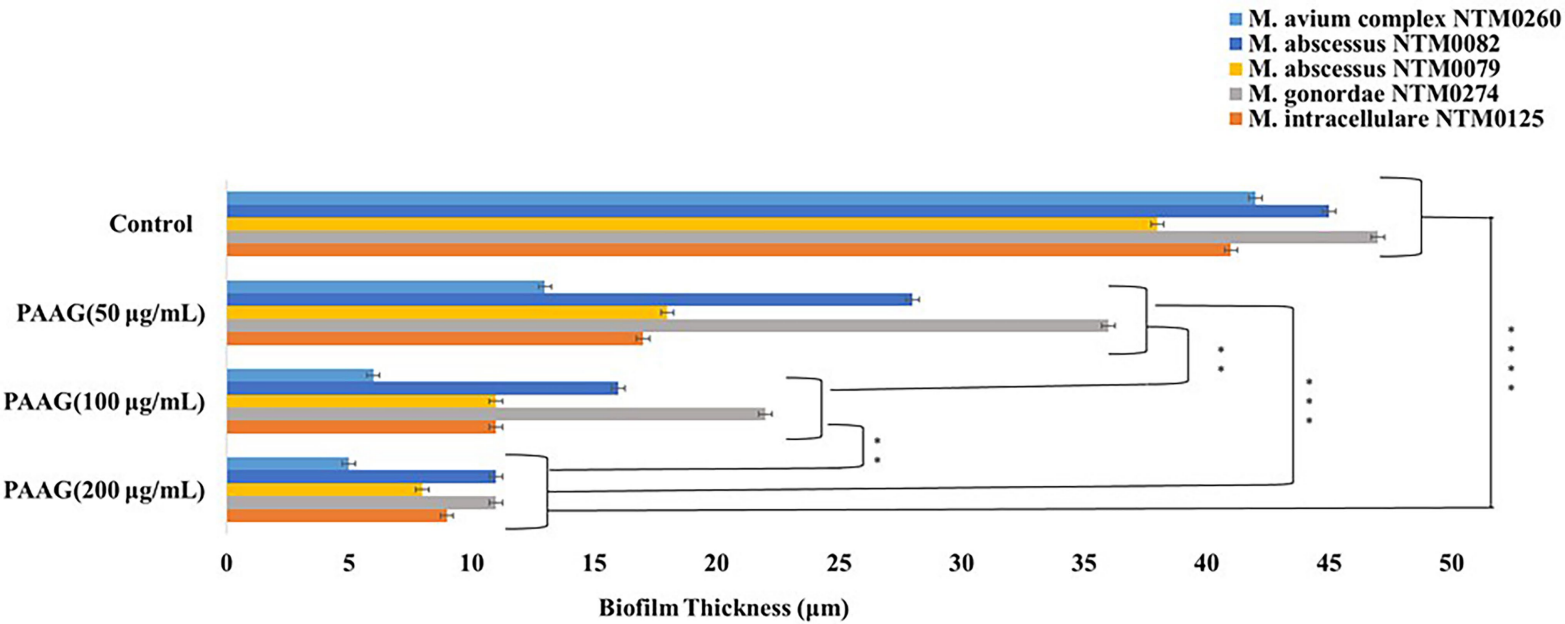
PAAG treatment resulted in a dose-dependent reduction of the NTM biofilm thickness. PAAG at concentrations of 50, 100, and 200 μg/ml led to significant reduction of the mature NTM biofilm thickness compared to the untreated controls. Statistical analysis was done using Dunnett’s multiple comparison tests compared to the untreated control. ** *p* < 0.01; *** *p* < 0.001; and **** *p* < 0.0001.

PAAG (200 μg/ml) was observed to significantly reduce NTM biofilm thickness, for all the strains tested, at depths <11 μm within 1 h of treatment. Five replicates (n) were used for each data point. Treatment with PAAG (200 μg/ml) resulted in a significant decrease (*p* < 0.001) in biofilm thickness compared to the 50 μg/ml PAAG treatment (*p* < 0.01). PAAG (200 μg/ml) was associated with a 75%–78% reduction in NTM biofilm thickness in 1 h, compared to the 23%–62% observed upon treatment with PAAG (50 μg/ml), in a strain dependent manner (Figure 8).

### Scanning Electron Microscopy of PAAG Treated Biofilms

**Figure 9:**
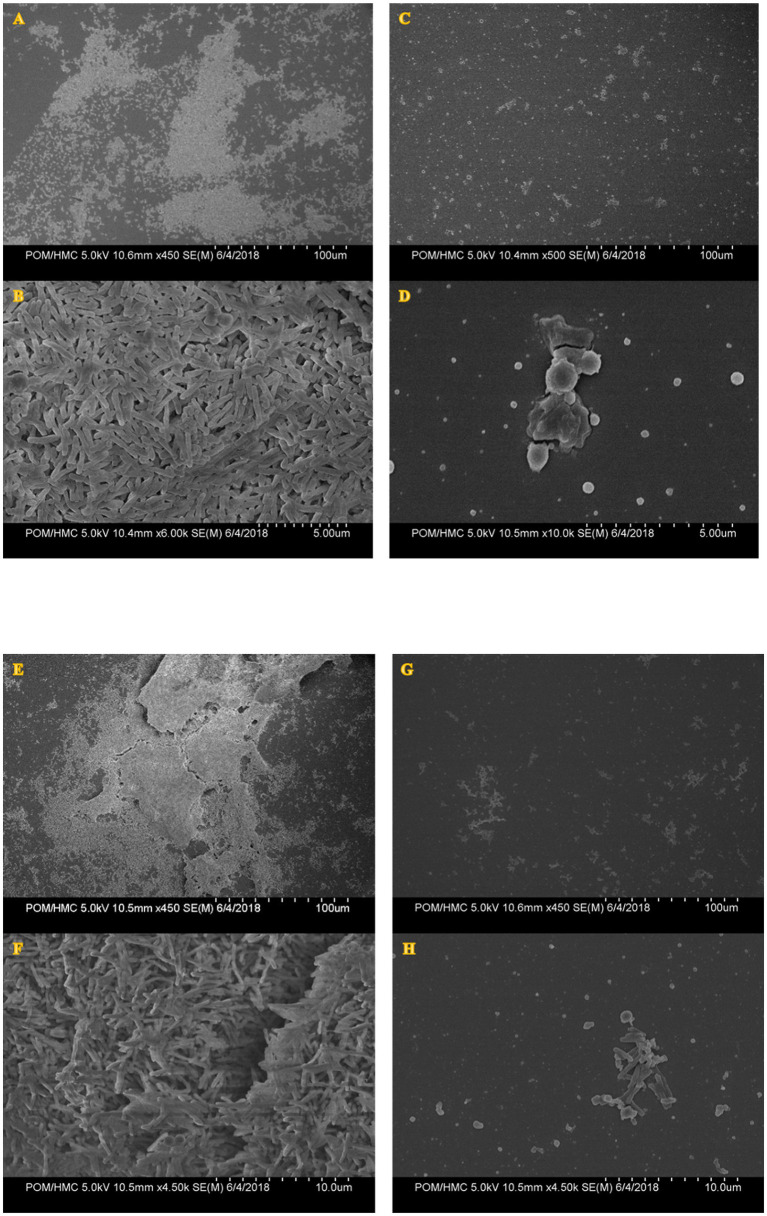
Scanning electron micrographs of MABSC (NTM 0079) **(A–D)** and MAC (NTM 0260) **(E–H)** biofilms before and after PAAG treatment. Biofilms were grown *in vitro* using the colony biofilm model for 4–12 days and treated with PAAG (200 μg/ml) for 1 h or left untreated (untreated control) prior to SEM imaging. Panels **(A**, **B**, **E**, **F)** are untreated controls. Panels **(C**, **D**, **G**, **H)** were taken post-treatment with PAAG for 1 h. Images shown are representative of five images taken for each sample (*n* = 5) from three independent experiments (*N* = 3).

A 1-h treatment of preformed biofilms with PAAG resulted in disruption of the biofilm structure formed by clinical isolates of *M. avium* and *M. abscessus* as shown in Figures 9C,D,G,H compared to the untreated control (Figures 9A,B,E,F). Untreated biofilms demonstrated a smoother surface with a “normal rod-like” shape (Figures 9A,B,E,F). PAAG was also observed to permeabilize the remaining bacterial cells with subsequent formation of cell debris accompanied by morphological differences in mycobacterial cell shape (Figures 9C,D,G,H).

## Discussion

This study examined the *in vitro* activity of PAAG against five clinical isolates of NTM (four RGM and one SGM) frequently associated with *CF* patient lung infections. The NTM strains tested include *M. avium* complex (MAC), *M. intracellulare,* and *M. abscessus* complex (MABSC), the most drug-resistant strains (O’Brien et al., 1987; Choudhri et al., 1995; Marras and Daley, 2002; Henry et al., 2004; Cassidy et al., 2009; Thomson, 2010) known to be common causative agents of pulmonary NTM disease. Exposure to PAAG permeabilized the bacterial cell wall of MAC and MABSC and eradicated metabolically inactive persister cells. PAAG appears unaffected by the growth phase, efflux pumps, or the metabolic state of bacteria.

Antibacterial profiling of the isolates confirmed that most of the strains tested were resistant to the antibiotics tested. The SGM strains tested were resistant to commonly used antibacterial drugs rifampicin and ethambutol. The RGM strain *M. abscessus* was resistant to rifampicin, ethambutol, amikacin, azithromycin, and ciprofloxacin. Environmental strains *M. gordonae* and *M. intracellulare*, commonly associated with *CF*, were found to be resistant to rifampicin, ethambutol, and azithromycin. Table 1 shows the anti-mycobacterial efficacy of PAAG at concentrations as low as 16 μg/ml against the drug-resistant strains of SGM like *M. avium complex*. At concentrations as low as 128–250 μg/ml PAAG showed antibacterial properties against rapidly growing, drug-resistant mycobacterial strain *M. abscessus* (Table 1). The general observation of innate drug resistance may be attributed to the unique structural organization of the NTM membrane into a bilayer of unusual lipids that reduces the influx of antibacterial agents (Bansal-Mutalik and Nikaido, 2014).

To better understand the antimicrobial efficacy of PAAG compared to standard of care antibiotics, time-kill curves were generated to determine whether PAAG’s effects were bactericidal or bacteriostatic. Ethambutol and azithromycin were bacteriostatic against the SGM MAC, resulting in <3 log reduction in the bacterial load compared to the starting inoculum (Figures 1F,G). Rifampicin was bactericidal against both RGM (MABSC) and SGM (MAC) strains tested (Figures 1A,E). Ethambutol and amikacin were bactericidal against RGM strains of MABSC (Figures 1B,C). However, the antibiotics failed to eradicate the viable bacteria in the culture, leaving behind a subpopulation of tolerant cells. PAAG, on the other hand, was bactericidal against all the NTM strains tested, eradicating the rapid and slow-growing NTM strains in a dose and time-dependent manner (Figures 1D,H). This rapid bactericidal activity appears to result from physical interactions between PAAG and NTM that support increased cell membrane permeability (Tang et al., 2010).

PAAG molecules have a positive charge distribution along the polysaccharide backbone, which is hypothesized to locally destabilize the bacterial membrane upon contact and facilitate increased membrane permeability. As predicted, PAAG treatment resulted in rapid permeabilization of both slow and rapid-growing NTM membranes, resulting in decreased membrane integrity and bacterial death (Figures 2A,B). Antibiotics showed inconsistent permeabilization of the NTM membranes and intermittent phases of quiescent periods where the membranes were stable were observed.

Since PAAG has been previously observed to depolarize the inner membrane of Gram-positive and Gram-negative bacteria, the study also assessed its ability to depolarize the NTM membrane using the fluorescent chemical 3,3-diethyloxacarbocyanine iodide (DiOC2; Narayanaswamy et al., 2017, 2018; Fernandez et al., 2019). The dye accumulates on the cell membrane and is sensitive to the polarization of membranes (Novo et al., 2000). Exposure of NTM to PAAG leads to rapid collapse in membrane potential, suggestive of the increase in membrane depolarization. A greater than 80% increase in RFU was observed within 1 h of treatment with PAAG, compared to the antibiotics tested (Figures 2C,D). Rifampicin and ethambutol were more effective in depolarizing the inner membranes of NTM compared to the other antibiotics tested and were more effective against the RGM than the SGM.

Antibiotic-induced persister cells have been shown to have a tolerance to clinically used antimicrobials, consistent with the observed persistence of infection in patients (Yam et al., 2020). Once the stress or antibiotic was removed, the persisters regrew in antibiotic-free media, as described by Balaban et al. (2019) in guidelines for research on antibiotic persistence. PAAG’s potential to target the bacterial membrane supports the investigation of its effect on the subpopulation of non-replicating, metabolically dormant cells called persisters. PAAG treatment resulted in eradicating the MABSC and MAC population within days of treatment (Figure 3). No regrowth was observed when the PAAG treated cultures were resuspended in PAAG free medium up to 7 or 21 days (Figure 3). Rifampicin was bactericidal against the RGM MABSC strain but not the SGM strain tested. Although the concentrations of rifampicin were bactericidal against the rapidly growing MABSC isolate, regrowth was observed when the cultures were resuspended in antibiotic-free medium. The biphasic killing pattern demonstrated by the other antibiotics tested on both RGM and SGM strains proves the existence of persister cells within the treated cultures. PAAG at 1X MIC was able to eradicate the NTM persisters formed due to antibiotic exposure in both RGM and SGM isolates tested. Furthermore, the addition of PAAG alongside the antibiotic treatment resulted in the complete eradication of the NTM persisters in addition to preventing regrowth in antimicrobial-free media, (Figure 5) signifying the efficacy of PAAG and lack of interference by antibiotics. The potential of PAAG to rapidly eradicate NTM persisters suggests a potential to reduce the spontaneous formation of persisters in patients that are highly recalcitrant to antimicrobial treatments.

Interestingly, PAAG was observed to disperse NTM biofilms formed by all the *CF* isolates tested, facilitating disruption and significant removal of NTM biofilms in a dose and time-dependent manner (Figure 6). PAAG rapidly penetrates NTM biofilms, permeabilizes bacteria, and decreases the thickness of the biomass (Figure 8). Increasing PAAG concentration from 100 to 200 μg/ml increased the permeabilization of the cells and reduced the thickness of the biomass. Significant biofilm disruption occurred within 10 min following treatment with 200 μg/ml PAAG compared to vehicle control, suggestive of its rapid interaction (Figure 2). The PAAG treated NTM biofilms, and vehicle controls were visualized by SEM (Figure 9) and CLSM (Figure 7). Visualization *via* CSLM microscopy post-LIVE/DEAD staining supported a better understanding of PAAG’s ability to disrupt and disperse the mature biofilms formed by these drug-resistant NTM strains. PAAG treated biofilms showed significantly lower amounts of live bacteria in addition to a substantial reduction in biofilm thickness compared to the untreated controls (Figures 7, 8). The SEM images following PAAG treatment helped further elucidate the mechanism and how PAAG disrupts the mature NTM biofilms by permeabilizing the bacteria and instigating morphological changes (Figure 9). Compared to the untreated controls, the PAAG treated biofilms appeared disrupted and dispersed when observed through SEM. Previous studies show PAAG targets *CF* mucus structures to displace divalent cations (Fernandez et al., 2019). Similar displacement of divalent cations in an extracellular polysaccharide (EPS) matrix would also disrupt the cohesion of the biofilm because of weakened electrostatic interactions (Garcia et al., 2022).

The current study shows PAAG is a potent inhibitor of mycobacterial growth with bactericidal activity against SGM and RGM clinical isolates. Exposure to PAAG resulted in rapid permeabilization of the outer membrane and a loss of membrane potential, indicating that membrane disruption is a critical component of its mechanism. The antibacterial activity against persister cells and drug-resistant NTM demonstrated by PAAG is supportive of further study of its potential to treat recalcitrant infections that require extended therapeutic intervention. Further development of PAAG for clinical use against NTM has the potential to significantly improve the treatment and outcomes for *CF* and non-*CF* patients with intractable infections.

## Data Availability Statement

The raw data supporting the conclusions of this article will be made available by the authors, without undue reservation.

## Author Contributions

VN: substantial contributions to the conception and design of the work, the acquisition, analysis, and interpretation of data for the work, and drafting the work and revising it critically for important intellectual content. AL: revised the work critically for important intellectual content. ST: substantial contributions in design and interpretation of data for the work, revising the work critically for important intellectual content, provide approval for publication of the content, and agree to be accountable for all aspects of the work in ensuring that questions related to the accuracy or integrity of any part of the work are appropriately investigated and resolved. SB and WW: revising the work critically for important intellectual content, provide approval for publication of the content, and agree to be accountable for all aspects of the work in ensuring that questions related to the accuracy or integrity of any part of the work are appropriately investigated and resolved. All authors contributed to the article and approved the submitted version.

## Conflict of Interest

The authors are employees and/or shareholders in Synedgen, Inc. These studies were funded by Synedgen, Inc. The funder Synedgen, Inc was not involved in the study design, collection, analysis, interpretation of data, the writing of this article or the decision to submit it for publication. SB, ST, and WW have ownership and patents affiliated with Synedgen, and SB and WW are board members. The potential conflicts noted have not impacted or influenced the findings of this manuscript.

## Publisher’s Note

All claims expressed in this article are solely those of the authors and do not necessarily represent those of their affiliated organizations, or those of the publisher, the editors and the reviewers. Any product that may be evaluated in this article, or claim that may be made by its manufacturer, is not guaranteed or endorsed by the publisher.
